# *Notes from the Field:* Circulating Vaccine-Derived Poliovirus Type 1 and Outbreak Response — Papua New Guinea, 2018

**DOI:** 10.15585/mmwr.mm6805a6

**Published:** 2019-02-08

**Authors:** Mathias Bauri, Amanda L. Wilkinson, Berry Ropa, Keith Feldon, Cynthia J. Snider, Abhijeet Anand, Graham Tallis, Liliane Boualam, Varja Grabovac, Tigran Avagyan, Mohammad S. Reza, Dessie Mekonnen, Zaixing Zhang, Bruce R. Thorley, Hiroyuki Shimizu, Lea Necitas G. Apostol, Yoshihiro Takashima

**Affiliations:** ^1^National Department of Health, Port Moresby, Papua New Guinea; ^2^Global Immunization Division, Center for Global Health, CDC; ^3^Global Polio Eradication Initiative, Geneva, Switzerland; ^4^World Health Organization, Geneva, Switzerland; ^5^Western Pacific Regional Office, World Health Organization, Manila, Philippines; ^6^World Health Organization Country Office, Port Moresby, Papua New Guinea; ^7^Victorian Infectious Diseases Reference Laboratory, Doherty Institute, Melbourne, Australia; ^8^National Institute of Infectious Diseases, Tokyo, Japan; ^9^Research Institute for Tropical Medicine, Muntinlupa, Philippines.

The last poliomyelitis cases reported in Papua New Guinea occurred in 1996. Papua New Guinea is one of 37 countries (or areas) of the World Health Organization Western Pacific Region that were certified free of indigenous wild poliovirus in 2000. On June 22, 2018, the National Department of Health confirmed an outbreak of poliomyelitis caused by circulating vaccine-derived poliovirus type 1 (cVDPV1) following isolation of genetically linked virus from a patient with paralysis and nonhousehold community contacts. The index patient was a boy aged 6 years from Lae, Morobe Province, with onset of paralysis on April 25 and history of having received 2 doses of Sabin oral poliovirus vaccine (OPV).* Genetic characterization of the isolate identified 14 nucleotide differences from the Sabin 1 strain in the VP1 coding region, suggesting circulation for >1 year. As of February 4, 2019, a total of 26 confirmed cases had been identified in nine of 22 provinces, including 19 in children aged <5 years, six in patients aged 5–14 years, and one in a patient aged 17 years. The most recent case onset was October 18, 2018 (Figure). Eighteen (69%) cases were linked to areas with large transient populations, including those near mines or plantations.

**FIGURE:**
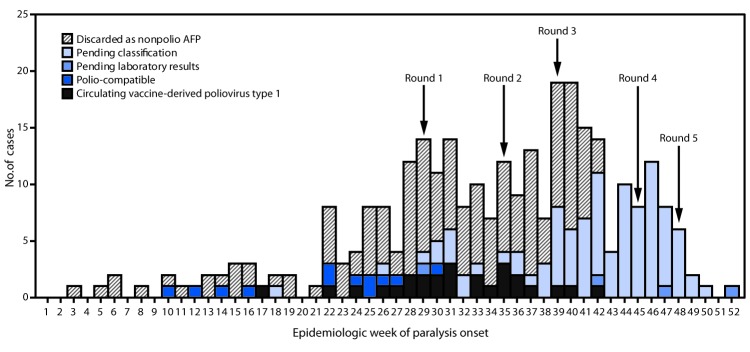
Number of acute flaccid paralysis (AFP) cases, by week* of paralysis onset, case classification,^†^ and SIA round^§^ — Papua New Guinea, 2018 **Abbreviation:** SIA = supplementary immunization activity. * Onset date was missing for 10 cases (two discarded as nonpolio AFP and eight pending classification). ^†^ Pending classification by Papua New Guinea’s National Polio Expert Committee. AFP cases pending classification have inadequate stool specimens (adequate = two stool specimens collected within 14 days of paralysis onset and ≥24 hours apart that arrive at a World Health Organization–accredited laboratory in good condition) from which no poliovirus was isolated. After committee review, these cases might be classified as polio-compatible or discarded as nonpolio AFP. Polio-compatible cases are AFP cases with inadequate specimens from which no poliovirus was isolated but in which there is polio-compatible residual paralysis at 60 days, death takes place within 60 days, or the case is lost to follow-up, and the cases are compatible with poliomyelitis based on available clinical information reviewed by the National Polio Expert Committee. ^§^ Shown are the start weeks for each of the five SIA rounds, during which bivalent (types 1 and 3) oral poliovirus vaccine was administered.

cVDPVs can emerge in underimmunized populations when Sabin vaccine poliovirus is extensively transmitted person-to-person and reverts to neurovirulence (*1*). Reported national administrative coverage for the third dose of OPV in infancy was 44% in 2017 and never exceeded 70% during 2006–2016 (*2*), with substantial subnational variation. The last previous national OPV vaccination campaign occurred in 2012.

The outbreak response included two subnational supplementary immunization activities (SIAs) (Round 1 and Round 2) (Figure) with bivalent OPV (containing OPV types 1 and 3), beginning July 16, 2018, and August 20 and targeting children aged <5 years in provinces with cVDPV1 cases or geographic or travel links to affected provinces. After cVDPV1 cases were detected in other provinces and in additional older children, two national SIAs were conducted (Round 3 and Round 4), targeting 3.26 million children aged <15 years. Reported administrative coverage^†^ was 93% for the nationwide SIA conducted beginning September 24 and 97% for the one beginning October 29. Beginning November 26, a third subnational SIA (Round 5) was conducted, targeting children aged <15 years. All SIAs faced logistical challenges requiring access to remote communities via helicopter, boat, and by foot.

Papua New Guinea was at risk for delayed detection of poliovirus because of an insufficient surveillance system for acute flaccid paralysis (AFP) and delays in seeking health care, often because of geographic inaccessibility. After the initial cVDPV1 case was detected, active AFP case-finding at health facilities was intensified. The annual national nonpolio AFP rate, a key indicator of surveillance sensitivity (*3*), was 7.0 per 100,000 persons aged <15 years in 2018 because of improved surveillance, compared with 0.8 in 2017.^§^ However, in 2018, adequate stool specimens^¶^ were received for <50% of AFP cases. After environmental sampling was established at three sites in Port Moresby, the largest city in Papua New Guinea, and two sites in Lae, the second-largest city, cVDPV1 was isolated from seven sewage samples in Port Moresby, beginning in September and most recently on November 6.

A December outbreak response assessment concluded that cVDPV1 transmission likely continues, given the dates of isolation of cVDPV1 from environmental surveillance and the most recent confirmed case. Additional SIAs are planned in Papua New Guinea in 2019. Because of the outbreak, on July 12, 2018, CDC issued a Level 2 Travel Health Notice recommending that all travelers to Papua New Guinea be fully vaccinated against polio. Before traveling to Papua New Guinea, adults who completed their routine polio vaccine series as children are advised to receive a single, lifetime adult booster of polio vaccine.

## Footnotes

* Before May 2016, all childhood vaccination with OPV was with trivalent (types 1, 2, and 3); since May 2016, vaccination has been with bivalent OPV (types 1 and 3) worldwide.

† Calculated as the number of doses administered divided by the estimated target population, and multiplied by 100.

§ A nonpolio AFP rate of ≥2 per 100,000 persons aged <15 years is considered sufficiently sensitive to detect a case of wild poliovirus or cVDPV if poliovirus is circulating.

¶ Adequate specimens are two stool specimens collected within 14 days of paralysis onset and ≥24 hours apart that arrive at a World Health Organization–accredited laboratory in good condition.
